# Intramuscular Cyst in the Vastus Intermedius Muscle: A Rare Presentation of a Hydatid Cyst

**DOI:** 10.7759/cureus.65248

**Published:** 2024-07-24

**Authors:** Manasa M Suryadevara, Ravishankar Patil, Gaurav V Mishra, Pratapsingh Parihar, Mounika Suryadevara, Devyansh Nimodia, Sheetal Shelar, Anshul Sood

**Affiliations:** 1 Radiodiagnosis, Jawaharlal Nehru Medical College, Datta Meghe Institute of Higher Education and Research, Wardha, IND; 2 Pulmonary Medicine, Ramaiah Medical College, Bengaluru, IND

**Keywords:** ultrasound (u/s), mri- magnetic resonance imaging, swelling, thigh mass, hydatid cyst

## Abstract

Echinococcosis, or hydatid disease, is a parasitic infection caused by a cestode from the Taeniidae family, mainly by Echinococcus multilocularis or granulosus. It is predominantly seen in the lungs or the liver. The hydatid disease rarely manifests as a palpable mass in the muscles. This study reports a case of a 70-year-old male who has presented with a swelling in the anterolateral aspect of his right upper thigh, which was progressive over the past two years. The swelling was initially painless and is now associated with pain. The clinical diagnosis of an abscess was suspected. The diagnosis of his swelling was later made as a hydatid cyst in a muscle of the thigh based on the imaging modalities, the ultrasound, and an MRI. The patient underwent surgical excision of the cyst, following which the diagnosis of a hydatid cyst was confirmed on the biopsy.

## Introduction

Echinococcosis, or hydatid disease, is caused by various species of Echinococcus, mainly granulosus and multilocularis. The organisms have both definitive and secondary hosts. The definitive hosts, like dogs, foxes, or wolves, pass eggs into the environment through excretion, and the intermediate hosts (humans, goats, cattle, sheep, camels, and horses) subsequently develop the disease when they ingest the eggs [1]. The lesion usually presents as a painless, slowly increasing mass and is mostly asymptomatic. The symptoms can occur when there is an increase in size [2]. The presenting symptoms can depend on the site and the growth of the lesion. The most commonly involved organs are the liver and lungs, followed by the brain. Other sites that are rarely involved include the bone, smooth and skeletal muscles, mediastinum, and viscera [1]. Hydatid cysts very rarely involve the musculoskeletal system. The incidence of isolated intramuscular hydatid cysts is 0.2 to 2.2% [3]. In this study, we present a case of an intramuscular thigh hydatid cyst in an old male patient, discussing the patient's clinical presentation, diagnosis, and treatment.

## Case presentation

A 70-year-old male presented with complaints of swelling over the right upper thigh on the anterolateral aspect, which has gradually increased in size for around two years. The swelling was initially painless and is now associated with pain. The pain is intermittent and intensifies with physical exertion. He is a known case of hypertension. 

**Figure 1:**
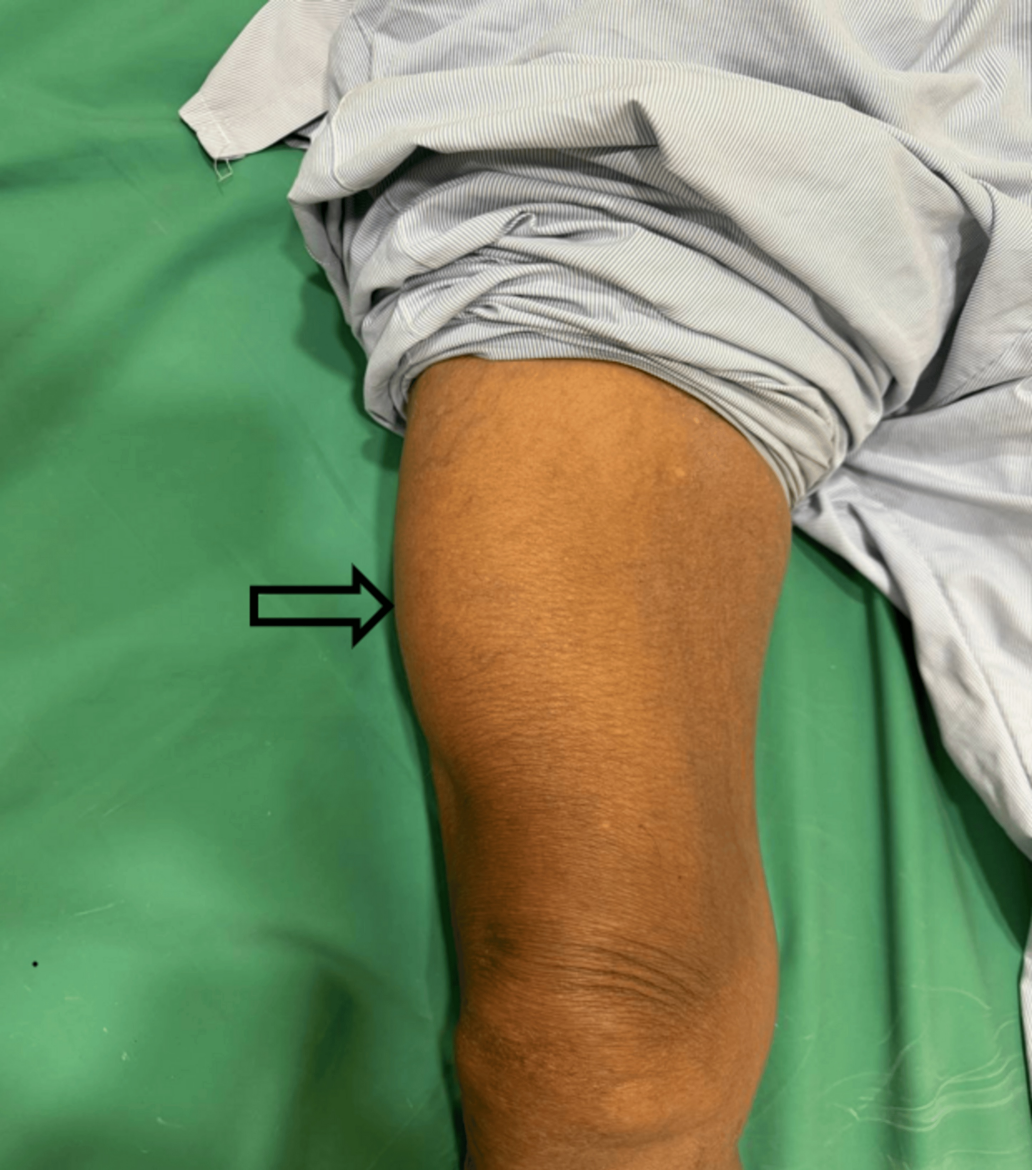
Swelling in the anterolateral aspect of the right thigh (arrow).

Examination showed normal vital signs. The local examination revealed a large oval palpable mass in the anterolateral aspect of the right thigh. The swelling measured about 8 x 4.5 cm (Figure 1).

**Figure 2:**
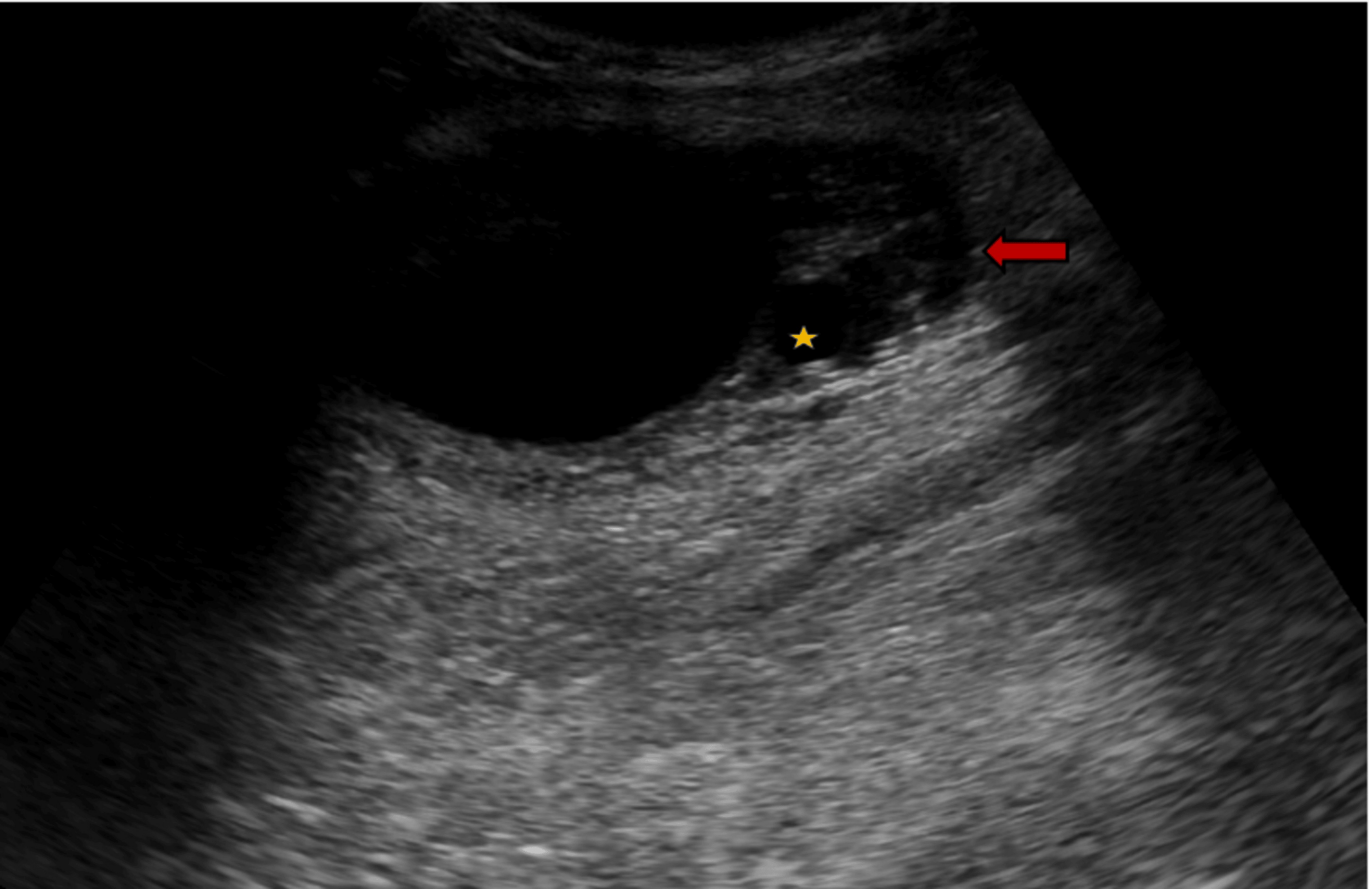
Ultrasound image showing well-defined oval cystic lesion (arrow) with multiple small cystic lesions (star) adjacent to it within the muscle.

It was firm, tender, and associated with raised temperature. There were no enlarged lymph nodes. A clinical diagnosis of an abcess has been made, and an ultrasound was advised. The patient was taken for an ultrasound, which showed a well-defined cystic mass in the intramuscular plane of the upper part of the thigh in its anterolateral aspect. The lesion measured about 7.5 x 4.7 cm. There were also multiple small cysts of various sizes adjacent to it, suggesting a hydatid cyst with multiple daughter cysts (largest, 10 x 8 mm) (Figure 2).

**Figure 3:**
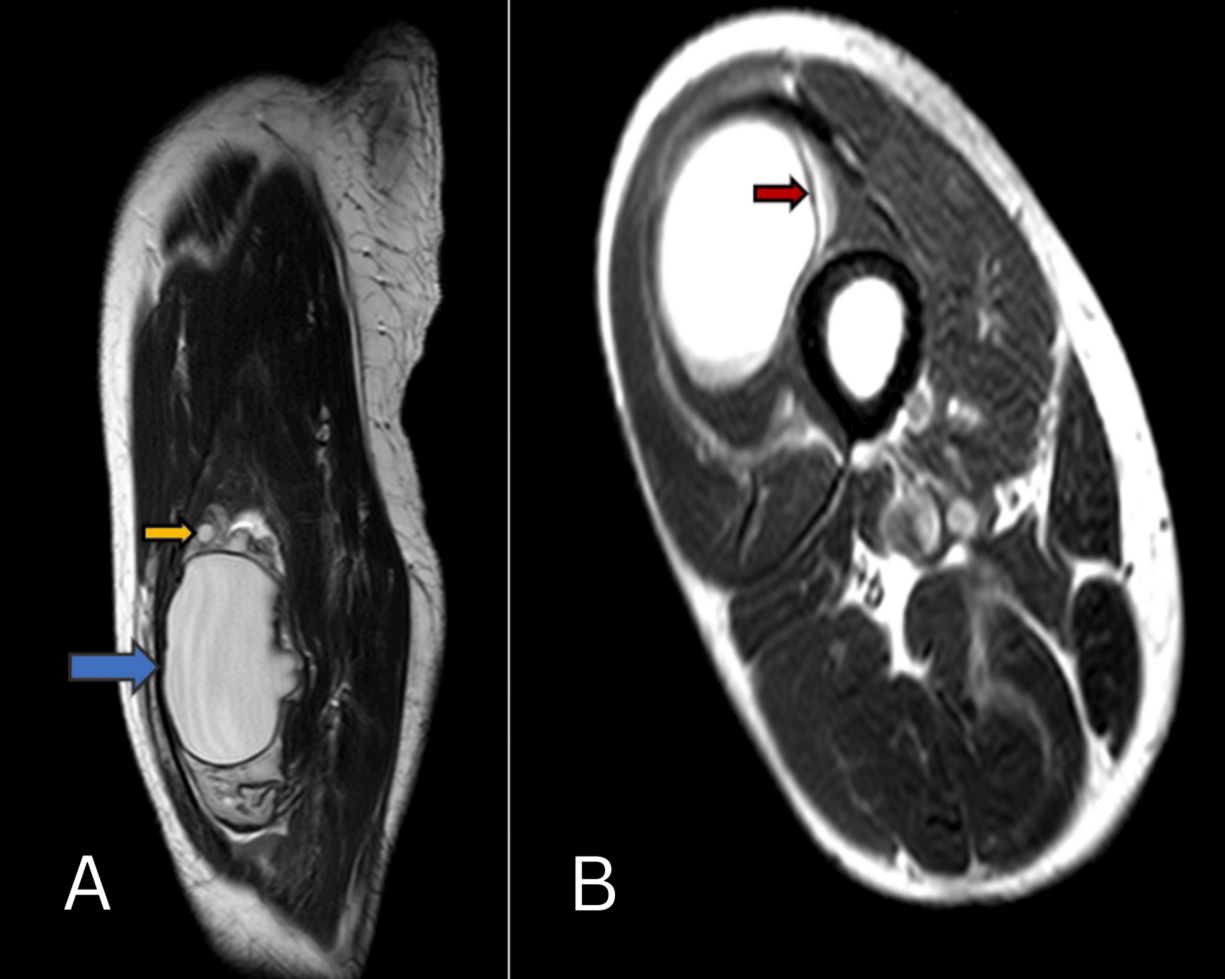
MRI T2-weighted A-sagittal and B-axial images show a well-defined cystic lesion in the vastus intermedius muscle (blue arrow) with a floating membrane within (red arrow) and with small cystic lesions adjacent to it (yellow arrow).

MRI revealed a well-defined, lobulated, oval cystic mass in the vastus intermedius muscle. The lesion measured about 8 x 4.9 cm. The lesion was seen involving the underlying periosteum of the femur. Multiple small cysts with homogenous content were also noted around the lesion (Figure 3). The MRI findings further confirmed the suspicion of a hydatid cyst. 

**Figure 4:**
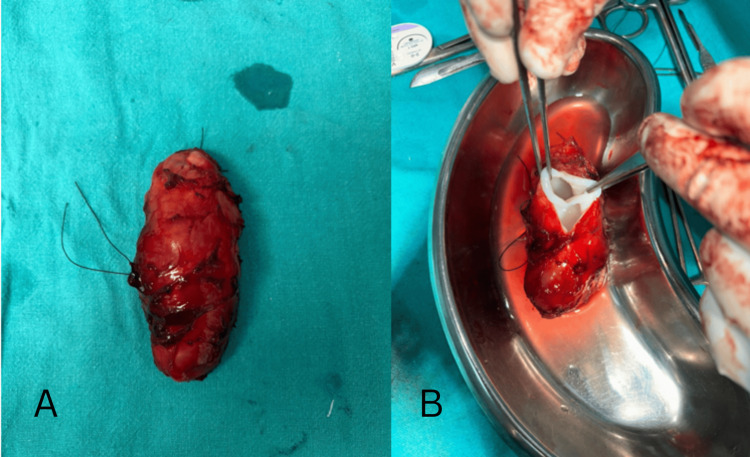
Post-op gross specimen. A: hydatid cyst measuring 8 x 5 cm; B: cut open section showing small daughter cysts seperated by septae

The patient then underwent a CT scan for the thorax and abdomen, which ruled out the involvement of other organs. Intraoperatively, under anesthesia, a lazy s-cut was made over the skin, overlying the mass in the right upper thigh, and swelling was identified beneath the rectus muscle. The plane was created around the lesion, and the mass was excised in toto (Figure 4), following which a wound wash was given using betadine and hydrogen peroxide (H2O2). The muscle layer and skin were closed.

The specimen was sent for histopathological examination, which showed a thick fibrous cyst wall, and within the cyst, germinal membrane and non-viable scolices were found, confirming the diagnosis of a hydatid cyst. The patient was prescribed 400 mg of albendazole twice daily. After two and five months of follow-up, the post-op recovery was uneventful.

## Discussion

Hydatid disease is a zoonotic infection caused by the species Echinococcus, mainly the granulosus and multilocularis. These organisms have both primary and secondary hosts. Dogs, foxes, and wolves form the definitive hosts, while cattle, humans, horses, and goats form the intermediate hosts. The definitive hosts excrete the eggs into the environment, which are then ingested by the intermediate hosts, subsequently developing the disease [1]. The most frequently involved sites are the liver and the lungs [4,5]. Other sites involved are the spleen, soft tissues, and bone [5]. Soft tissue involvement is rare and is usually secondary to another source in the body. The general sites for muscle involvement in hydatid include the neck, hip, trunk, arms, and thigh, which is likely a result of the rich vascularity of these sites [6].

The exceptional character of hydatid cysts in any site of the body is that the organism can stay dormant for a long while without the causation of specific symptoms. It is either incidentally diagnosed or when it causes pressure symptoms, making the patient seek out medical attention [1]. The lesion in the presented case caused a symptomatic thigh swelling.

It is crucial to diagnose hydatid disease before surgery. The sensitivity of the serology tests depends on the type of hydatid, where the majority (around 90%) of the hepatic hydatids test positive. In contrast, most hydatids in other body parts test negative [7]. Sonography is the imaging modality most often used for soft tissue swellings, which gives information about the location of the swelling and the characteristics of fluid within it [8]. However, the preferred imaging method for atypical hydatid diseases is MRI, which provides excellent information about the structure and the relationship of the soft tissues. In the present case, the ultrasound sonography (USG) helped with the initial diagnosis, suggesting the hydatid disease, and the MRI confirmed the diagnosis [9].

Many factors are involved in determining the management of hydatid disease, like the location, severity of the symptoms, and complications, if any. Complete surgical excision is the recommended treatment for the hydatid in muscle. Measures should be taken not to cut open the cavity to avoid spillage of the contents and minimize the recurrence. Using the antihelminthic drugs postoperatively will help reduce the rate of recurrence [6,10].

## Conclusions

Hydatid disease is a parasitic infection whose diagnosis is challenging as it mimics other conditions. The incidence of hydatid disease involving the thigh is an infrequent manifestation that can be diagnosed by MRI typically and managed definitively with complete surgical excision.
